# An unusual presentation of adenoid cystic carcinoma of the breast with metastatic disease in the clavicle

**DOI:** 10.1259/bjrcr.20160119

**Published:** 2017-01-28

**Authors:** Thomas Edward Glover, Ryan Butel, Cara Manmeet Bhuller, Emma Louise Senior

**Affiliations:** ^1^Department of Medicine, West Suffolk NHS Foundation Trust, Suffolk, UK; ^2^Department of Histopathology, West Suffolk NHS Foundation Trust, Suffolk, UK; ^3^Department of Surgery, West Suffolk NHS Foundation Trust, Suffolk, UK; ^4^Department of Radiology, West Suffolk NHS Foundation Trust, Suffolk, UK

## Abstract

Adenoid cystic carcinoma (ACC) of the breast is a rare subtype of invasive breast cancer. Prognosis is excellent with low rates of recurrence and metastatic disease compared with other triple-negative forms of breast carcinoma and other non-breast forms of ACC. We present a case of a 63-year-old female with metastatic disease in the clavicle 13 years after excision of the breast primary. Metastasis to bone is rare, and this is the first case described in the clavicle. There are no specific radiological features of breast primaries but imaging usually reveals a circumscribed mass, often without microcalcifications. Histology is similar to that of non-breast forms of ACC. Mastectomy or wide local excision is curative in virtually all cases without lymph node involvement. However, as our case demonstrates, the presence of bone pain with a history of ACC of the breast should prompt musculoskeletal imaging. Discussion at a multidisciplinary team meeting is essential for accurate diagnosis.

Breast cancer is an umbrella term for a number of different types of carcinoma of the breast. Adenoid cystic carcinoma (ACC) of the breast is a rare subtype of invasive breast cancer, making up less than 0.1% of all cases.^1–4^ Rarer still is metastatic spread of this cancer to bone, with only 11 cases reported in the literature to date.^5^ This subtype is typically negative for oestrogen (ER), progesterone (PR) and human epidermal growth factor (HER2) receptors but in contrast to other triple-negative subtypes, it rarely metastasizes and 10 year survival can be as good as 90–100%.^5^ This case report presents a rare complication of ACC of the breast in the form of metastatic spread to the clavicle.

## Case report

**Figure 1.**
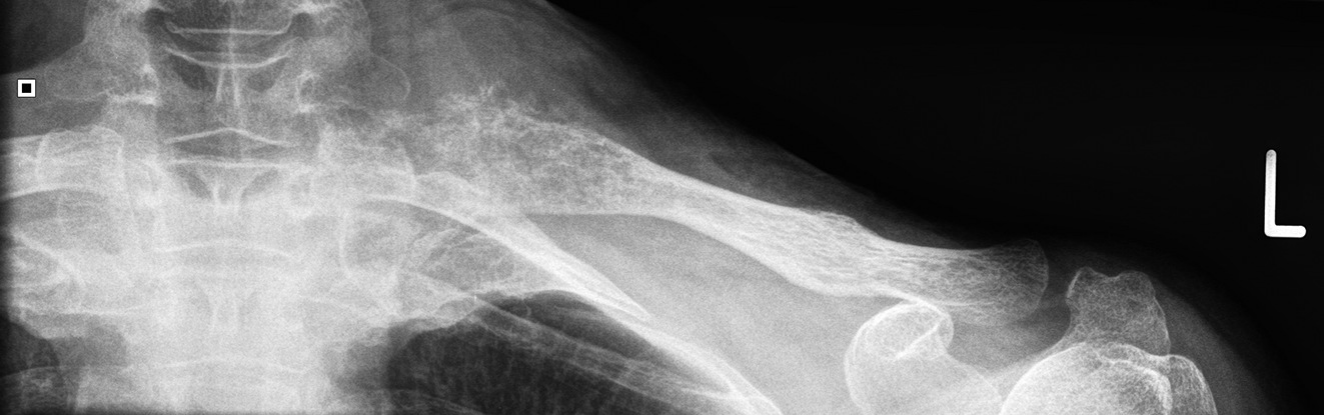
Radiograph showing destructive bony lesion of the medial left clavicle.

**Figure 2.**
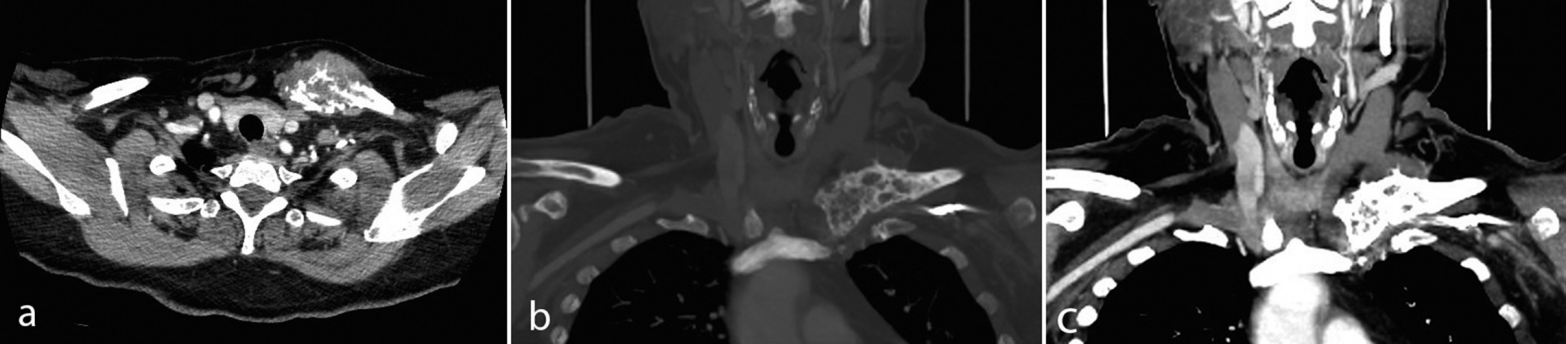
(a) Axial CT scan with i.v. contrast medium showing an expansile, predominantly lytic lesion of the medial left clavicle with associated tissue mass using soft tissue windows. (b) Coronal CT scan with i.v. contrast medium demonstrating the mass at the medial left clavicle using bone windows. (c) Coronal CT scan with i.v. contrast medium demonstrating the mass at the medial left clavicle using soft tissue windows.

**Figure 3.**
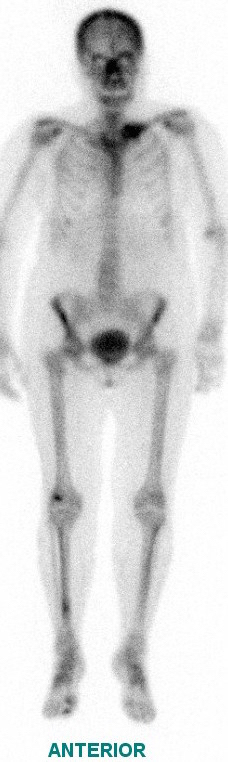
Bone scintigraphy showing an area of increased activity in the left clavicle correlating with the CT findings. An area of increased activity was also noted in the distal shaft of the right tibia and was suspicious of metastatic disease. This area, however, was later proven to be a false positive.

**Figure 4.**
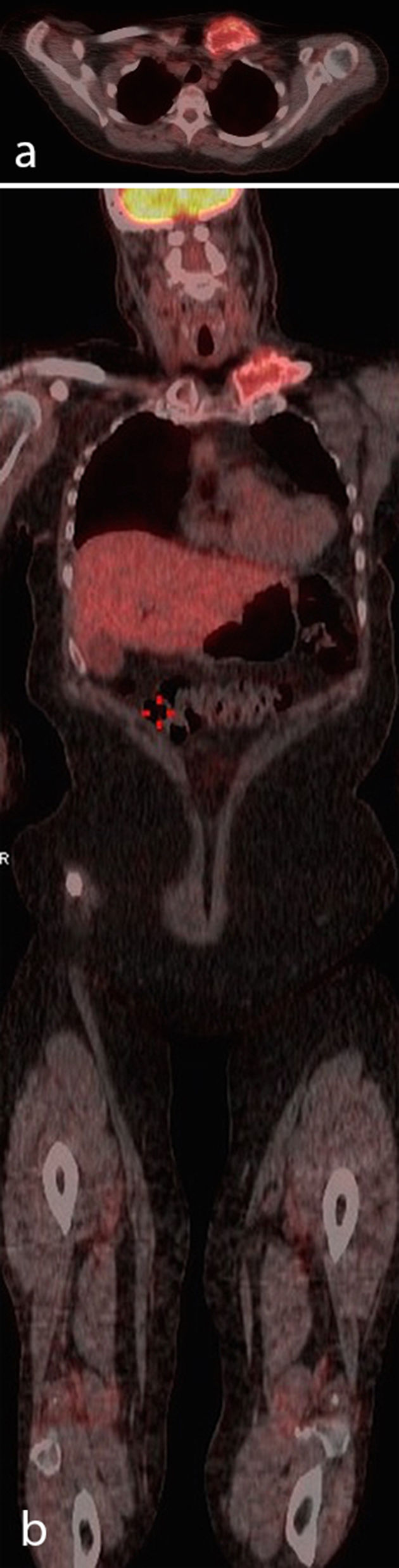
(a) Axial FDG-PET/CT slice showing bony destruction of the medial end of the clavicle with associated soft tissue and demonstrating moderate tracer uptake (SUV_max_ = 4.7). (b) Coronal FDG-PET/CT slice showing the medial left clavicle lesion. FDG-PET, fludeoxyglucose-positron emission tomography; SUV, standardized uptake value.

**Figure 5.**
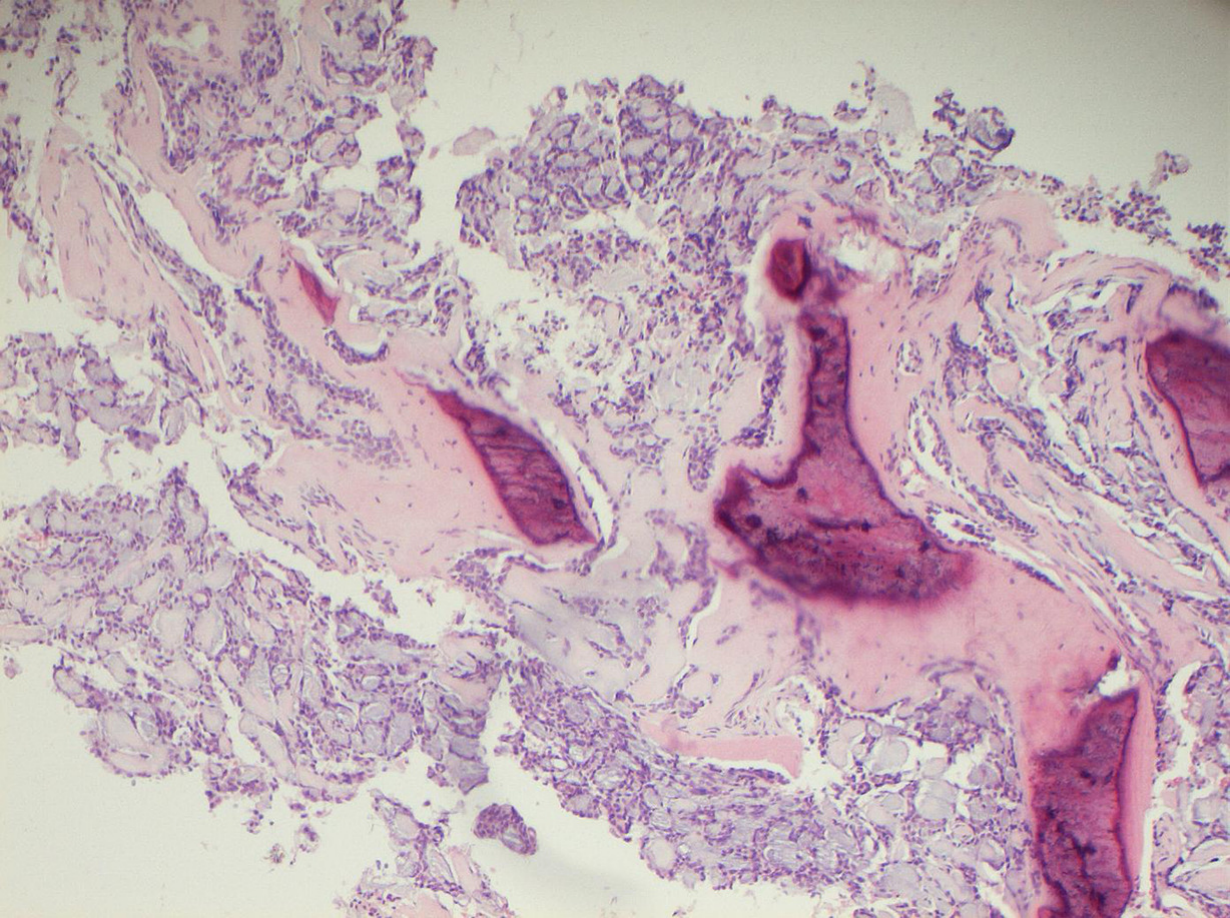
Photomicrograph showing a core biopsy of the bone invaded by adenoid cystic carcinoma (hematoxylin & eosin 100× magnification).

**Figure 6.**
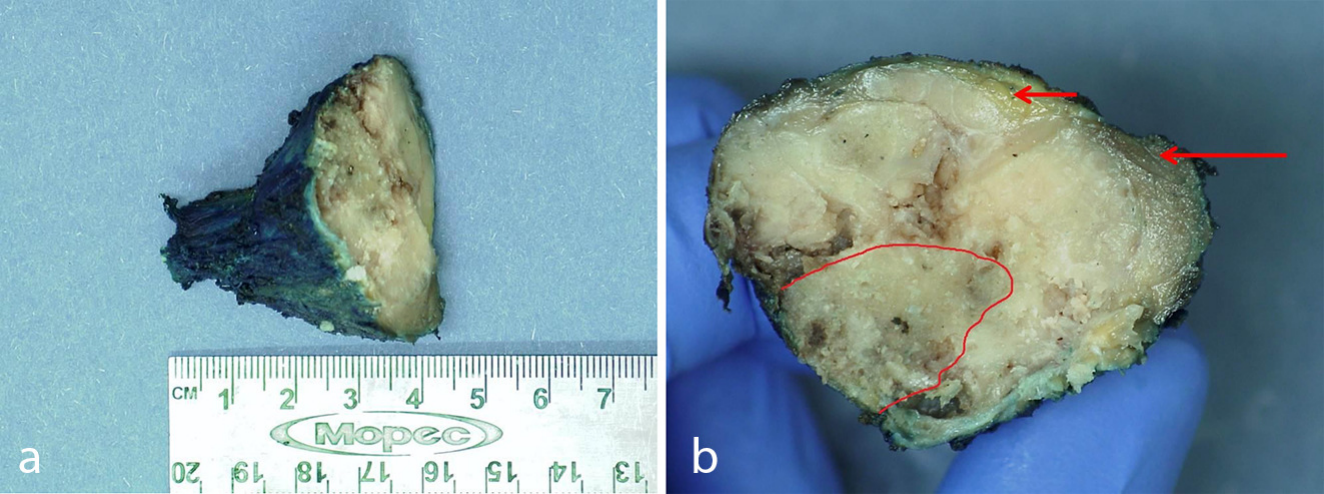
Resection specimen of clavicle with tumour. (a) Small portion of normal clavicle (left) with expansile tumour, transected for sampling (right). (b) Transverse section showing probable residual clavicle (red border) involved by tumour that has expanded into skeletal muscle (long arrow) and fat (short arrow).

A 63-year-old female presented in 2016 with pain in her left clavicle while out walking her dog. She had a history of grade I, node negative, triple receptor negative ACC of the left breast, which was treated with mastectomy in 2003. Eight years later she was diagnosed with a new primary tumour in the contralateral (right) breast. This was a grade III, 3/17 node positive, ER positive/HER2 negative invasive ductal carcinoma, which was treated with mastectomy, axillary node clearance, chemoradiotherapy and letrozole (Femara, Novartis, UK). The patient had been disease free up to the latest presentation in 2016. The clavicular pain initially settled spontaneously but recurred and prompted further investigations. Clinical examination revealed a mass involving the left clavicle measuring 5 cm in transverse diameter. A radiograph of the left clavicle showed evidence of an expansile destructive lesion at its medial end (Figure 1). This finding, in addition to the soft tissue invasion, was confirmed on CT (Figure 2), bone scintigraphy (Figure 3) and PET-CT (Figure 4). Given the atypical nature of this lesion on a background of breast cancer, a bone biopsy was performed. Histology showed cores of bone invaded by a tumour with a biphasic pattern of epithelial and myoepithelial cells forming cribriform and tubular structures containing eosinophilic secretions (Figure 5). There were no solid areas to indicate high grade disease and no perineural invasion was seen. Immunohistochemical staining was positive for keratin 7 but negative for ER/PR/HER2, keratin 20, gross cystic disease fluid protein-15 and thyroid transcription factor-1. The histology from the original breast carcinoma was reviewed and this showed a similar histomorphological pattern. In the absence of an additional radiological primary site, the bone lesion was reported as primary metastatic ACC in keeping with the previously diagnosed left breast cancer and recommended for multidisciplinary team discussion. The patient underwent surgical resection of the lesion and clavicle. Macroscopic examination showed an expansile tumour in the clavicle with probable extension into soft tissues (Figure 6). In concordance with the biopsy, histology revealed a tumour with classic ACC features invading bone and confirmed soft tissue extension. The patient is now undergoing adjuvant radiotherapy to the area.

## Discussion

ACC of the breast is a well-recognized but rare primary invasive malignancy of the breast accounting for less than 0.1% of all breast malignancies.^2,3,6^ It typically occurs in females aged between 50 and 63 years. ACC of the breast is generally considered relatively indolent, with favourable prognosis despite its basal-like molecular phenotype and triple negative hormone receptor status. Compared with other more common forms of non-mammary ACC (e.g. salivary gland and bronchial) there is a low rate of perineural invasion and local recurrence.^6^

Presentation is usually of a unifocal well circumscribed palpable mass found most often in the upper outer quadrant or subareolar region.^7^ Pain and nipple retraction also occur. Just like other types of breast cancer, mammography and ultrasonography are the mainstay imaging techniques. Although variations may occur, ACC is often smooth and lobulated with well-circumscribed margins or simply subtle architectural distortions on mammogram. The former is commoner in less dense breast tissue, while denser breast tissue may obscure the lobulated mass presenting as a false-negative mammogram.^5^ Microcalcifications are rare and ultrasound may show a heterogeneous, hypoechoic and circumscribed mass.^7^ MRI studies of ACC often show benign, irregular masses that show rapid gadolinium enhancement. Diagnosis is primarily via core biopsy but diagnosis with fine needle aspiration has also been made.^8^ Histology shows a similar pattern to other forms of ACC, such as in the salivary gland, with a biphasic growth pattern of epithelial and myoepithelial cells. Often periodic acid-Schiff positive hyaline material may be present within the luminal spaces. Immunohistochemistry usually reveals the epithelial component to be positive for keratin 7 and negative for p63, while the myoepithelial cells generally show positivity for smooth muscle actin, calponin and p63. Staining for ER, PR, HER2 and gross cystic disease fluid protein-15 is usually negative throughout.^5^

Largely, ACC of the breast is an indolent cancer that rarely metastasizes or recurs. Lymph node involvement is rare but the incidence increases with increasing tumour size.^9^The most common site for distant spread is lung,^10^ but spread to kidney, brain and bone has also been reported.^5,10–12^ Pulmonary metastases have been noted as late as 9 years from disease onset, confirming slow disease progression.^5^ Of the handful of cases describing metastasis to bone in the literature, none specify which part of the bone is most affected, making our report unique.

Our case showed the classic histological features of ACC, and upon review had a similar morphology and immunohistochemical profile to the original primary tumour in the left breast. In the absence of another radiologically identified primary site, breast was the only obvious source. In line with the indolent behaviour of the tumour, our patient has had 13 disease-free years.

## Conclusions

The potential for recurrence and/or metastasis from ACC of the breast is rare and excision is usually curative in node negative patients. Bone metastasis is very rare but can, as our case demonstrates, occur even after a long disease free period (here 13 years). As such, the presence of bone pain in a patient with a history of ACC of the breast should prompt musculoskeletal imaging. Review of any previous breast pathology and discussion at a multidisciplinary meeting is essential.

## Learning points

1. ACC of the breast is indolent and rarely metastasizes.
2. Bone pain and a history of ACC of the breast should prompt musculoskeletal imaging.
3. Mammography and ultrasonography are the main imaging techniques used for ACC of the breast.
4. Recurrences and or metastases can occur many years after the original tumour diagnosis.

## Consent

Written informed consent for the case to be published (incl. images, case history and data) was obtained from the patient(s) for publication of this case report, including accompanying images.
